# Impact of Multi-Causal Transport Mechanisms in an Electrolyte Supported Planar SOFC with (ZrO_2_)_x−1_(Y_2_O_3_)_x_ Electrolyte

**DOI:** 10.3390/e20060469

**Published:** 2018-06-16

**Authors:** Gerardo Valadez Huerta, Vincent Flasbart, Tobias Marquardt, Pablo Radici, Stephan Kabelac

**Affiliations:** Institut für Thermodynamik, Gottfried Wilhelm Leibniz Universität Hannover, Callinstraße 36, D-30167 Hannover, Germany

**Keywords:** entropy production, SOFC, electrochemistry, fuel cell, solid-state ionics

## Abstract

The calculation of the entropy production rate within an operational high temperature solid oxide fuel cell (SOFC) is necessary to design and improve heating and cooling strategies. However, due to a lack of information, most of the studies are limited to empirical relations, which are not in line with the more general approach given by non-equilibrium thermodynamics (NET). The SOFC 1D-model presented in this study is based on non-equilibrium thermodynamics and we parameterize it with experimental data and data from molecular dynamics (MD). The validation of the model shows that it can effectively describe the behavior of a SOFC at 1300 K. Moreover, we show that the highest entropy production is present in the electrolyte and the catalyst layers, and that the Peltier heat transfer is considerable for the calculation of the heat flux in the electrolyte and cannot be neglected. To our knowledge, this is the first validated model of a SOFC based on non-equilibrium thermodynamics and this study can be extended to analyze SOFCs with other solid oxide electrolytes, with perovskites electrolytes or even other electrochemical systems like solid oxide electrolysis cells (SOECs).

## 1. Introduction

Fuel cells are an alternative technology for power generation [1]. High temperature solid oxide fuel cells (SOFCs) have high efficiency and are, together with the polymer-electrolyte membrane fuel cell (PEMFC), the most promising type [2,3]. Some of the main advantages of SOFCs are the high tolerance to CO and their flexibility with the purity of the H_2_ needed, in comparison to PEMFCs [4,5]. SOFC systems with pre- or internal reforming can work with common fuels in the actual infrastructure and also work in a future hydrogen network [6,7,8]. Depending on the electrolyte material and thickness, SOFCs are operated at a temperature between 700 °C and 1100 °C [9,10].

Consistent simulation data is beneficial considering the effort needed to obtain reliable results for different electrolytes through experimental research [11,12]. Many 2D-models or 3D-models of SOFCs exist in the literature [13,14,15,16,17,18,19], but only few are founded within the theory of non-equilibrium thermodynamics (NET) [20]. This theory includes possible coupled transport mechanisms driven by multiple gradients and does not claim mono-causal transport mechanisms as the classical kinetic equations [21,22,23]. Coupled transport mechanisms are; e.g., the Peltier effect—where a gradient in the electric potential drives a heat flux. The classical Fourier equation, used in many simulation models, only accounts for a heat flux due to a temperature gradient [24,25]. The coupling effects are small in many technical systems and neglecting them still gives reliable simulation results [26]. Since most simulation models have empirical parameters to be fitted to the individual application, a deficit in the kinetic equation does not become apparent immediately. It results in “effective” transport coefficients. A well-known case is the simulation of multi-component diffusion with Fick’s law instead of using the Maxwell–Stefan equations [27,28].

In NET [22,23] the fluxes $J_{i}$ of extensive thermodynamic properties are assigned to more than one generalized force $X_{j}$ [21]. The force $X_{j}$ is a gradient of an intensive property like temperature, chemical potential, etc. For the fluxes $J_{i}$, we can write the following phenomenological equations:

(1) $$J_{i}\approx \underset{j}{\sum }L_{ij}X_{j},$$

where $L_{ij}$ are the phenomenological coefficients, also called Onsager-coefficients or conductivities. This approximation is the result of a Taylor expansion of the entropy in dependency on the deviations from equilibrium of all variables and the assumption that all processes occur near equilibrium [29]. Only in this case, the Onsager relations for the phenomenological coefficients $L_{ij}=L_{ji}$ apply [30]. The entropy production rate $\dot{\sigma }$ is given by the fluxes and the corresponding forces:

(2) $$\dot{\sigma }=\underset{i}{\sum }J_{i}X_{i}.$$

This fundamental relationship between the local entropy production rate $\dot{\sigma }$ and the fluxes is extremely valuable for understanding dissipative phenomena and for possibly improving the efficiency of the cell by lowering the entropy production.

A 1D SOFC-model dealing with the transport mechanisms across the cell and using NET is given by S. Kjelstrup and D. Bedeaux [20]. In this model, empirical coefficients are used to calculate the conductivities $L_{ij}$ and to describe the bulk-phases and the catalyst layers CLs. The assumption of infinite surfaces and a small heat conductivity in their model approach leads to temperature jumps in the CLs. However, the authors do not calculate the entropy production rate. The studies of A. Sciacovelly and V. Verda [31,32], as well as the recent studies of J. J. Ramírez-Minguela et al. [33,34], deal with the 3D calculation of the entropy production rate in a SOFC. They only use empirical equations and do not conduct any validation. Other studies regarding the entropy production are limited to the system level [35].

Since the validation of all details in the typical standard simulation approach for a SOFC is not yet completed [36,37], it is the aim of this paper to compare standard kinetic equations against the more general NET approach. Especially for electrochemical systems showing increasingly strong gradients due to very thin functional layers, it seems advisable to check the validity of mono-causal kinetic equations. In this study, we conduct experiments on a single SOFC and develop a 1D-model of the cell in steady state. We evaluate the cell experimentally with electrochemical impedance spectroscopy (EIS) and measure the voltage (E)-current(j)-characteristic. The model is based on NET, except for the CLs, where we assume them to be isotherm and calculate the reaction rate using Butler–Volmer kinetics. For an overall simulation model based on the NET-approach all conductivities $L_{ij}$ need to be known [38]. These coefficients can be estimated from known (effective) standard transport coefficients [20,39,40], for example:

(3) $$L_{qq}=\frac{\lambda }{{\left(k_{B}\beta \right)}^{2}}+\frac{\kappa \pi^{2}}{k_{B}\beta },$$

where the conductivity $L_{qq}$ describes the heat transport in a solid conductor, λ the heat conductivity, κ the electronic conductivity and π the peltier coefficient. Furthermore, $\beta ={\left(k_{B}T\right)}^{-1}$ is the reciprocal of the temperature and $k_{B}$ the Boltzmann constant ($1.3806\times 10^{-23}\;J/K$). As the measured transport coefficients depend significantly on the exact individual nature of the sample; e.g., the grain size and boundaries [41,42]—care must be taken when using relations like Equation (3). To overcome these problems, the direct calculation of the conductivities can be done using MD [43,44,45].

We believe that the processes across the cell are the most relevant to give insights of the coupled mechanisms and, thus, we limit our study to the spatial dimension perpendicular to the cell area. The coefficients to describe the electrolyte are taken from our previous MD study [40]. These values agree with the experimental data and are calculated from a single “experiment”. Furthermore, we can evaluate Equation (1) without any transformation. Unfortunately, the values are given only for 1300 K and, thus, we limit our study to this temperature.

The novelty of our study lies mostly on the following features:

- We expand the gas diffusion layers’ (GDLs’) NET models of S. Kjelstrup and D. Bedeaux [20] accounting for diffusion.
- We give a detailed description of the transport mechanisms in YSZ electrolytes based on NET using the phenomenological coefficients calculated with MD.
- Our NET model is validated with our own experimental data.
- We discuss in detail the influence of the coupled mechanisms and calculate each contribution to the entropy production rate in each layer.

## 2. Materials and Methods

### 2.1. Materials and Experimental Methods

We conduct all measurements with an Evaluator-HT test facility from FuelCon AG, Germany. We use a SOFC of type KeraCell II manufactured by KERAFOL GmbH, Germany. The KeraCell II is an electrolyte supported cell (ESC) with an 8YSZ electrolyte and an electrolyte thickness of $1.6\times 10^{-4}\;m$. The NiO/GDC anode and the ScSZ/LSM cathode GDLs are $0.4\times 10^{-4}\;m$ thick. We conduct our measurements after a first commissioning of the cell. Here, the first step is to heat the cell to 1123 K. The heating process is done with a 1 K/min ramp function in order to achieve a homogenous temperature field across the cell. After heating, the temperature of the cell is held at 1123 K for several hours before the anode material is reduced to Ni/GDC.

**Figure 1:**
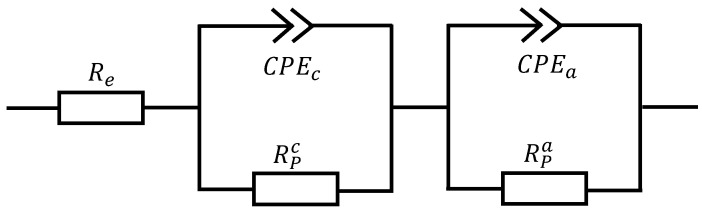
Equivalent electronic circuit.

We conduct our EIS-measurements with a ModuLab XM ECS from the company AMETEK GmbH at atmospheric pressure and different oven temperatures (1023 K, 1073 K, 1123 K, 1173 K and 1300 K). The EIS-measurements are galvanostatic with a current amplitude of $\pm 280\times 10^{-3}\;A$ in a frequency range of 0.1 Hz to 100 kHz. We choose the equivalent circuit given in Figure 1. We use three elements in series. The first element is a resistance to describe the ohmic losses from the electrolyte. In the second and the third element, we use a resistance and a constant phase element (CPE) in parallel to describe the losses in the catalyst layers (CLs). We fit our impedance spectra with the equivalent circuit using the software XM-studio of the analyzer. From this fitting, we get the electrolyte resistance $R^{e}$ and the polarization resistances $R_{P}^{i}$.

We conduct an extra measurement of the E-j-characteristic of the cell with an oven temperature of 1300 K. This measurement is galvanostatic in a range of 0 to $8000\;A/m^{2}$.

### 2.2. Theoretical Methods

#### 2.2.1. SOFC-System

**Figure 2:**
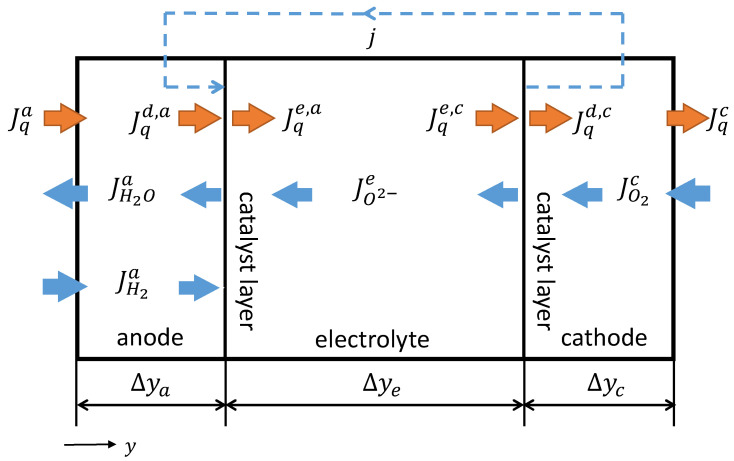
Solid oxide fuel cell (SOFC) including the electric current density j, all heat fluxes $J_{q}^{i}$ and all molar fluxes $J_{k}^{i}$. The spatial coordinate y is defined perpendicular to the cell area.

We define y as the spatial coordinate perpendicular to the SOFC active area and divide the cell in the following regions: the anode GDL, the anode CL, the electrolyte, the cathode CL and the cathode GDL. The anode GDL has a thickness of $\Delta y_{a}$, the electrolyte a thickness of $\Delta y_{e}$ and the cathode diffusion layer a thickness of $\Delta y_{c}$. In Figure 2, the resulting system including all the heat and molar fluxes is depicted. Since we only consider one spatial direction, the fluxes can be written as scalars and not as vectors.

The oxygen molar flux $J_{O_{2}}^{c}$ coming from the gas channel flows through the cathode diffusion layer and into the CL. Here, an oxygen atom reacts with two electrons to build oxygen anions during the oxygen reduction reaction (ORR):

(4) $$1/2\;O_{2}\left(g\right)+2e^{-}⇄O^{2-}.$$

The oxygen anions are then transported through the solid oxide electrolyte. In this work, we study only YSZ-electrolytes. The doping of Y_2_O_3_ in ZrO_2_ to form YSZ results in vacancies [46]. This defect formation can be described by the following reaction in Kröger–Vink notation:

(5) $$Y_{2}O_{3}\overset{ZrO_{2}}{↔}2Y^{\prime }+V_{O}^{••}+3O_{O}^{x},$$

with the electro-neutrality condition $\left[Y^{\prime }\right]=2\left[V_{O}^{••}\right]$. The anion flux $J_{O^{-2}}^{e}$ is transported through these defects in the opposite direction of the electric current density j carrying two negative electric charges. From the gas channel through the anode GDL, the hydrogen molecules are transported as part of a molar flux $J_{H_{2}}^{a}$ to the anode CL, where they react with the anions. From this hydrogen oxidation reaction (HOR), water and two free electrons result:

(6) $$H_{2}\left(g\right)+O^{2-}⇄H_{2}O\left(g\right)+2e^{-}.$$

The electrons flow opposite to j from the anode to the cathode in an external electronic circuit.

We define all heat fluxes in the direction of the spatial coordinate y. The heat flux $J_{q}^{a}\left(y\right)$ flows through the anode GDL. At the CL, the heat flux $J_{q}^{a}\left(\Delta y_{a}\right)$ is equal to $J_{q}^{d,a}$. Due to the half-cell reaction, heat may be released at the CL, so that the heat $J_{q}^{e,a}$ flowing from the anode to the electrolyte is not necessarily equal to $J_{q}^{d,a}$. From then on, the heat $J_{q}^{e}\left(y\right)$ with $J_{q}^{e}\left(\Delta y_{a}\right)=J_{q}^{d,a}$ flows through the electrolyte and, at the CL, $J_{q}^{e}\left(\Delta y_{a}+\Delta y_{e}\right)$ is equal to $J_{q}^{e,c}$. Again, the heat released from the cathode reaction results in a non-equality between $J_{q}^{e,c}$ and $J_{q}^{d,c}$. Finally, the heat flux $J_{q}^{c}\left(y\right)$ with $J_{q}^{c}\left(\Delta y_{a}+\Delta y_{e}\right)=J_{q}^{d,c}$ is transported further through the cathode diffusion layer.

We only consider a H_2_,H_2_O-ideal gas mixture at the anode side and an O_2_,N_2_-ideal gas mixture at the cathode side. The thermodynamic state point is given by T=1300 K and $p=p^{o}=1\;\mathrm{bar}$, and we only consider steady state conditions.

#### 2.2.2. Mathematical Description of the GDLs

Our start point is the energy balance for the anode GDL and the cathode GDL:

(7) $$0=\frac{dJ_{q}^{a}}{dy}+j\frac{d\phi }{dy}+J_{H_{2}}^{a}\frac{dH_{H_{2}}}{dy}-J_{H_{2}O}^{a}\frac{dH_{H_{2}O}}{dy},$$

(8) $$\;0=\frac{dJ_{q}^{c}}{dy}+j\frac{d\phi }{dy}-J_{O_{2}}^{c}\frac{dH_{O_{2}}}{dy},$$

where ϕ describes the electric potential. Heat $J_{q}^{i}$ and electric power j·ϕ are transported through the GDLs, while the gas components k enter or leave them with the molar enthalpy $H_{k}$. The molar enthalpy of ideal gases is only dependent on the temperature and can be expressed differentially as $dH_{k}=C_{p,k}\left(T\right)dT$, where $C_{p,k}\left(T\right)$ is the molar isobaric heat capacity of species k. We define all molar fluxes relative to the spatial direction (see Figure 2) and, considering the component balances $2J_{H_{2}}^{a}=-2J_{H_{2}O}^{a}=j/F$ and $-4J_{O_{2}}^{c}=j/F$, we get:

(9) $$0=\frac{dJ_{q}^{a}}{dy}+j\left[\frac{d\phi }{dy}+\frac{1}{2F}\left[C_{p,H_{2}}\left(T\right)+C_{p,H_{2}O}\left(T\right)\right]\frac{dT}{dy}\right],$$

(10) $$0=\frac{dJ_{q}^{c}}{dy}+j\left[\frac{d\phi }{dy}+\frac{1}{4F}C_{p,O_{2}}\left(T\right)\frac{dT}{dy}\right],$$

where F is the Faraday constant (96,485 C/mol). All thermodynamic properties for the ideal gases are calculated with the equations of Kabelac et al. [47]. To describe the fluxes and thermodynamic forces we use the following kinetics [20]:

(11) $$J_{q}^{i}=-\lambda^{i}\frac{dT}{dy}+\pi^{i}j$$

(12) $$j=-\frac{\pi^{i}}{r^{i}}\frac{1}{T}\frac{dT}{dy}-\frac{1}{r^{i}}\frac{d\phi }{dy},$$

(13) $$J_{k}^{i}=-D_{k}\frac{dc_{k}}{dy},$$

where the upper script i=a,c denotes the anode or the cathode, $r^{i}$ the area specific resistance and $D_{k}$ the effective diffusion coefficient of species k. The derivation of Equations (11) and (12) using non-equilibrium thermodynamics can be found in the work of S. Kjelstrup and D. Bedeux [20].

The area specific resistance $r^{i}$ is described by following the Arrhenius equation:

(14) $$r^{i}/T=r_{0}^{i}\cdot e^{\frac{E_{A,r}^{i}}{RT}}.$$

Here, $E_{A,r}^{i}$ is the activation energy and $r_{0}^{i}$ the pre-exponential factor. To give Equation (14) theoretical support, we firstly assume that all phenomenological coefficients $L_{ij}$ may have an Arrhenius behavior. The coefficient $L_{\phi \phi }$, which describes the electronic transport, is related to the electronic conductivity κ as follows:

(15) $$F^{2}L_{\Phi \Phi }^{i}=\kappa^{i}T.$$

Equation (15) can be derived from Equation (1) following the steps given in our previous work [48]. As a consequence, the following Arrhenius equation arises:

(16) $$\kappa^{i}T=\kappa_{0}^{i}\cdot e^{-\frac{E_{A,r}^{i}}{RT}}.$$

This result is supported by experimental data [49]. The electronic conductivity behaves inversely proportionally to the area specific resistance; i.e., $\kappa \sim 1/r^{i}$ and the proportionality factor is of mere geometrical nature. Therefore, Equation (14) follows directly from Equation (16) having the same activation energy, different pre-exponential factor, and inverse temperature dependency.

As can be taken from Equation (11) and from Equation (12), the Peltier coefficient $\pi^{i}$ not only describes the heat transported due to charge transport, but also the contribution of a temperature gradient to the effective electric resistance.

The effective diffusion coefficient in Equation (13) also comprises multicomponent diffusion [24]. The Dusty gas model should give a better approximation than the one implemented in our model, because it accounts Knudsen diffusion [38]. However, following the discussion of R. Suwanwarangkul et al. [27], our model may also give satisfactory results within the simulation conditions (see below). We integrate Equation (13) to describe the concentration profile of hydrogen $c_{H_{2}}$ and water $c_{H_{2}O}$ at the anode GDL and oxygen $c_{O_{2}}$ at the cathode GDL:

(17) $$c_{H_{2}}\left(y\right)=c_{H_{2}}^{0}-\frac{jy}{2FD_{H_{2}}},$$

(18) $$c_{H_{2}O}\left(y\right)=c_{H_{2}O}^{0}+\frac{jy}{2FD_{H_{2}O}},$$

(19) $$c_{O_{2}}\left(y\right)=c_{O_{2}}^{0}-\frac{j}{4FD_{O_{2}}}\left(y_{c}-y\right).$$

Here, we use the component balances, the superscript 0 denotes the gas channel and $y_{c}=\Delta y_{a}+\Delta y_{e}+\Delta y_{c}$. Finally, we calculate the entropy production rate from Equation (2) as follows:

(20) $${\dot{\sigma }}^{i}=-J_{q}^{i}\frac{1}{T^{2}}\frac{dT}{dy}-j\frac{1}{T}\frac{d\phi }{dy}-R\underset{k}{\sum }J_{k}^{i}\frac{1}{c_{k}}\frac{dc_{k}}{dy},$$

where R is the ideal gas constant (8.3144 J/(molK)). From the Equations (13) and (17)–(19), a concentration dependent term is given for the anode and for the cathode:

(21) $$\underset{k}{\sum }J_{k}^{a}\frac{1}{c_{k}}\frac{dc_{k}}{dy}=-{\left(\frac{j}{2F}\right)}^{2}\left[\frac{1}{c_{H_{2}}D_{H_{2}}}+\frac{1}{c_{H_{2}O}D_{H_{2}O}}\right],$$

(22) $$\underset{k}{\sum }J_{k}^{c}\frac{1}{c_{k}}\frac{dc_{k}}{dy}=-{\left(\frac{j}{4F}\right)}^{2}\frac{1}{c_{O_{2}}D_{O_{2}}}.$$

#### 2.2.3. Mathematical Description of the CLs

We assume constant temperature at the CL and its surroundings, but with a jump in the electric potential and the heat flux. To describe the potential jumps we begin by applying the Nernst-Equation to calculate the open circuit voltage (OCV) $\Delta \phi_{0}^{i}$ for the anode half reaction (4) and for the cathode half reaction (6):

(23) Δϕ0a=12F[GH2Oo(T)−GH2o(T)+RTln(xH2ORxH2R)],

(24) Δϕ0c=12F[0.5·GO2o(T)+RT2ln(xO2R)],

where Gio describes the standard molar free enthalpy and the superscript R denotes the CLs. These equations result from the electrochemical equilibrium condition assuming $2F\Delta \phi_{0}^{i}={\tilde{\mu }}_{O^{2-}}-2{\tilde{\mu }}_{e^{-}}$, where ${\tilde{\mu }}_{k}$ is the electrochemical potential of the charged species k. The mole fractions $x_{k}^{R}$ are calculated from Equations (17) to (19) evaluated at $y^{R}=\Delta y_{a}$ for H_2_ and H_2_O and at $y^{R}=\Delta y_{a}+\Delta y_{c}$ for O_2_, and using the transformation $x_{k}^{R}=c_{k}\left(y^{R}\right)RT/p$. We define following Butler–Volmer equations for the ORR (4) and the HOR (6):

(25) $${\dot{\xi }}_{r}^{i}=\frac{j_{0}^{i}}{2F}\left(e^{-\frac{\alpha^{i}2F}{RT}\eta^{i}}-e^{\frac{\left(1-\alpha^{i}\right)2F}{RT}\eta^{i}}\right),$$

The rate of reaction ${\dot{\xi }}_{r}^{i}$ is imposed by the current density j, which determines the number of charges to be transferred per time unit. We can define $j=2F{\dot{\xi }}_{ORR}^{c}$ and $j=-2F{\dot{\xi }}_{HOR}^{a}$ from a charge balance. For each reaction, an anodic and a cathodic barrier must be overcome, which are described by exponential terms. The charge transfer coefficient $\alpha^{i}$ determines the proportion of anodic or cathodic losses to the total losses, and the over-voltages $\eta^{i}$ play the role of an activation energy. The exchange current density $j_{0}^{i}$ describes the current, which in equilibrium flows equally in both directions of the half reaction. A more detailed discussion, can be found in [50]. We calculate $j_{0}^{i}$ with an approach from Yonekura et al. [51]:

(26) $$j_{0}^{a}={\left(x_{H_{2}}^{R}\right)}^{0.41}{\left(x_{H_{2}O}^{R}\right)}^{0.4}\gamma^{a}e^{\frac{E_{A,j_{0}}^{a}}{RT}},$$

(27) $$j_{0}^{c}={\left(x_{O_{2}}^{R}\right)}^{0.3}\gamma^{c}e^{\frac{E_{A,j_{0}}^{c}}{RT}},$$

where $\gamma^{i}$ are pre-exponential factors. The exchange current density $j_{0}^{i}$ is related to the experimentally measurable polarization resistance $R_{P}^{i}$ as follows:

(28) $$j_{0}^{i}=\frac{RT}{2R_{P}^{i}F}.$$

The potentials at the reaction layers are given by:

(29) $$\phi^{e,a}=\phi^{d,a}-\Delta \phi_{0}^{a}-\eta^{a},$$

(30) $$\phi^{d,c}=\phi^{e,c}+\Delta \phi_{0}^{c}+\eta^{c}.$$

To calculate the heat fluxes, we use the entropy balances for each CL:

(31) $$J_{q}^{e,a}=J_{q}^{d,a}-T\frac{j}{2F}\left(S_{H_{2}O}\left(T\right)-S_{H_{2}}\left(T\right)-S_{O^{2-}}\left(T\right)+2S_{e^{-}}\left(T\right)\right)+T{\dot{S}}_{irr}^{a},$$

(32) $$J_{q}^{d,c}=J_{q}^{e,c}-T\frac{j}{2F}\left(S_{O^{2-}}\left(T\right)-\frac{1}{2}S_{O_{2}}\left(T\right)-2S_{e^{-}}\left(T\right)\right)+T{\dot{S}}_{irr}^{c}.$$

Here, $S_{k}$ is the molar entropy of species k, $S_{irr}^{i}$ the area specific entropy production rate. From the literature [39], we assume that $S_{O^{2-}}=42\;J/\left(\mathrm{molK}\right)$ and $S_{e^{-}}=1\;J/\left(\mathrm{molK}\right)$ . Finally, we can write with the overvoltage $\eta^{i}$ from Equation (25) the following equation:

(33) $${\dot{S}}_{irr}^{i}=-\eta^{i}\cdot \xi_{r}^{i}/T.$$

#### 2.2.4. Mathematical Description of the Solid Oxide Electrolyte

To calculate the fluxes, we can write from Equation (1) following set of phenomenological equations:

(34) $$J_{q}^{e}/k_{B}=-L_{qq}\frac{d\beta }{dy}-\underset{k}{\sum }L_{qk}\cdot \beta \cdot \frac{d\mu_{k}}{dy}-L_{q\varphi }\cdot \beta \cdot F\cdot \frac{d\varphi }{dy},$$

(35) $$J_{k}^{e}/k_{B}=-L_{kq}\frac{d\beta }{dy}-\underset{k}{\sum }L_{kk}\cdot \beta \cdot \frac{d\mu_{k}}{dy}-L_{k\varphi }\cdot \beta \cdot F\cdot \frac{d\varphi }{dy},$$

(36) $$j/\left(k_{B}F\right)=-L_{\varphi q}\frac{d\beta }{dy}-\underset{k}{\sum }L_{\varphi k}\cdot \beta \cdot \frac{d\mu_{k}}{dy}\;-L_{\varphi \varphi }\cdot \beta \cdot F\cdot \frac{d\varphi }{dy},$$

where φ the electrostatic potential in YSZ. The current density j describes the flux of electrons transported along with the ionic fluxes $J_{k}^{e}$ with k=Z,Y,O for $Zr^{4+}$, $Y^{3+}$ and $O^{2-}$. The conductivities $L_{kk}$ are given by the auto correlation function (ACF) of the micro ionic fluxes [40] and $L_{\varphi \varphi }$ by the current ACF [45]. Due to polarization, the micro ionic fluxes may not necessarily be proportional to j, resulting in coefficients with completely different natures. The coupled effect between the electron and the ionic transport is then given by the conductivities $L_{k\varphi }$. For 1300 K, no cation diffusion in YSZ occurs; i.e., $L_{ZZ}=L_{YY}=0$ [52]. Furthermore, Valadez Huerta et al. [46] compare their classical MD calculations of the Coulomb energy in YSZ with *ab-initio* calculations and do not find any substantial differences, concluding that polarization effects can be neglected. With these assumptions, we can write $J_{O^{2-}}^{e}=j/\left(z^{*}F\right)$ and

(37) $$J_{q}^{e}/k_{B}=-L_{qq}\frac{d\beta }{dy}-L_{qO}\cdot \beta \cdot \frac{d\mu_{O^{-2}}}{dy}-L_{q\varphi }\cdot \beta \cdot F\cdot \frac{d\varphi }{dy},$$

(38) $$J_{O^{2-}}^{e}/k_{B}=-L_{Oq}\frac{d\beta }{dy}-L_{OO}\cdot \beta \cdot \frac{d\mu_{O^{-2}}}{dy}-L_{O\varphi }\cdot \beta \cdot F\cdot \frac{d\varphi }{dy},$$

(39) $$j/\left(k_{B}z^{*}F\right)=-L_{\varphi q}\frac{1}{z^{*}}\frac{d\beta }{dy}-L_{\varphi O}\cdot \frac{\beta }{z^{*}}\cdot \frac{d\mu_{O^{-2}}}{dy}-L_{\varphi \varphi }\cdot \frac{\beta }{z^{*}}\cdot F\frac{d\varphi }{dy}$$

with the valency $z^{*}=-2$. Equations (38) and (39) are equivalent. Equating the coefficients and using the Onsager relations $L_{O\varphi }=L_{\varphi O}$ and $L_{q\varphi }=L_{\varphi q}$, we get $L_{\varphi \varphi }={\left(z^{*}\right)}^{2}L_{OO}$, $L_{\varphi O}=z^{*}L_{OO}$ and $L_{\varphi q}=z^{*}L_{Oq}$. As a result, only the conductivities $L_{OO}$ and $L_{Oq}=L_{qO}$ are necessary to describe the anionic conduction and heat transport. Using the definition of the electric potential $\phi ={\tilde{\mu }}_{O^{2-}}/\left(z^{*}F\right)$ with ${\tilde{\mu }}_{O^{2-}}=\mu_{O^{-2}}+z^{*}F\cdot \varphi$, two independent phenomenological equations arise:

(40) $$J_{q}^{e}=-L_{qq}\frac{1}{T^{2}}\frac{dT}{dy}-L_{qO}\frac{z^{*}F}{T}\frac{d\phi }{dy},$$

(41) $$J_{O^{2-}}^{e}=-L_{Oq}\frac{1}{T^{2}}\frac{dT}{dy}-L_{OO}\frac{z^{*}F}{T}\frac{d\phi }{dy}.$$

We can write for the entropy production rate in analogy to Equation (20)

(42) $${\dot{\sigma }}^{e}=-J_{q}^{e}\frac{1}{T^{2}}\frac{dT}{dy}-J_{O^{2-}}^{e}\frac{z^{*}F}{T}\frac{d\phi }{dy}$$

and for the energy balance

(43) $$0=\frac{dJ_{q}^{e}}{dy}-J_{O^{2-}}^{e}z^{*}F\frac{d\phi }{dy}.$$

#### 2.2.5. Computational Details

We conduct our calculation with MatLab^®^ Version R2017a. We solve the equations consecutively from the anode to the cathode using the Runge–Kutta (4,5) method [53]. We assume as initial conditions T(0)=1300 K and ϕ(0)=0 V. To reproduce the isothermal operational modus, we iterate numerically using the Newton–Raphson method. Thereby, the heat flux $J_{q}$ is a variable with a start value of $1000\;W/m^{2}$ until the temperature at $y=\Delta y_{a}+\Delta y_{e}+\Delta y_{c}$ reaches T(0) with an accuracy of $10^{-34}\;K$.

The parameters used to describe the GDLs and the CLs are given in Table 1. The parameters needed to describe the exchange current density $j_{0}^{i}$ in Equations (26) und (27) are derived directly from experiments (see next section). In the case of the electrolyte ($\Delta y^{e}=160\times 10^{-6}\;m$), we analyze different YSZ compositions and take the values for the phenomenological coefficients from our previous work (see Table 2) [40].

## 3. Results and Discussion

### 3.1. EIS Measurements

**Figure 3:**
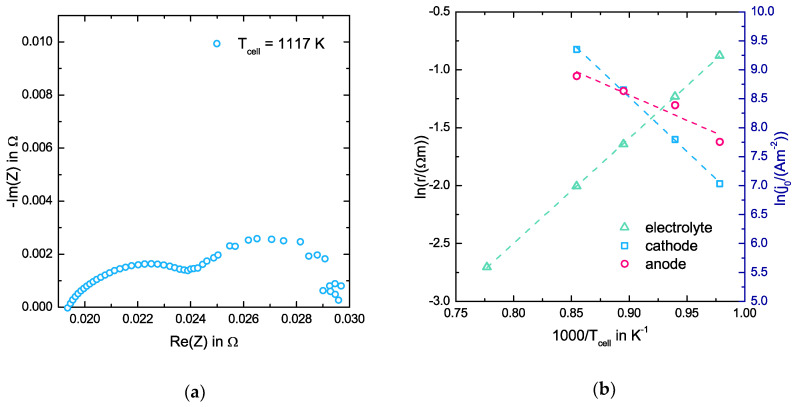
(**a**) Impedance spectrum at a cell temperature of 1117 K and (**b**) Arrhenius plot for the specific resistance r for the electrolyte and the exchange current density $j_{0}$ for the anode and the cathode.

We plot exemplarily in Figure 3a the impedance spectrum of the cell at a cell temperature of 1117 K. The measured cell temperature is not equal to the oven temperature due to heat losses. For the impedance spectrum in Figure 3, we calculate for the CPE at the anode an angle of 60.33° and at the cathode an angle of 83.52° despite the apparent circular progression of the curve. Therefore, our assumption of CPEs instead of perfect capacities is more accurate. In Figure 3b, we plot the Arrhenius plot for the specific resistance $r^{e}$ and the exchange current densities $j_{0}^{c,0}$ and $j_{0}^{a,0}$. From these Arrhenius plots, we get the pre-exponential factors $r_{0}^{e}=9.28\left(27\right)\times 10^{-5}\;\Omega m$, $\gamma^{a}=24\left(37\right)\times 10^{6}\;A/m^{2}$ and $\gamma^{c}=1.82\left(67\right)\times 10^{11}\;A/m^{2}$, and the activation energies $E_{A,r}^{e}=0.7816\left(65\right)e\;J$, $E_{A,j_{0}}^{c}=1.62\left(10\right)e\;J$ and $E_{A,j_{0}}^{a}0.75\left(43\right)e\;J$, where e is the elementary charge ($1.6022\times 10^{-19}\;C$). Here, the deviation describes the error due to the linear regression with a 95% confidence interval. The noise in the current results in a poorly defined spectrum at low frequencies; i.e., a low linearity and a high error in the values for the anode CL. The specific resistance $r^{e}$ at a cell temperature of 1300 K is 0.0622(40) Ωm. The value calculated from the coefficient $L_{OO}$ for YSZ08 is 0.0561(71) Ωm. The relative deviation between both values is only ~10% indicating that our approach effectively describes the electrolyte ionic conductivity. At this point it should be mentioned that the effective resistance may also include a Peltier contribution given by the temperature gradient and the phenomenological coefficient $L_{oq}$ (see Equation (40)), which is not included in the coefficient $L_{OO}$. This will be discussed later on in more detail.

### 3.2. Model Validation

**Figure 4:**
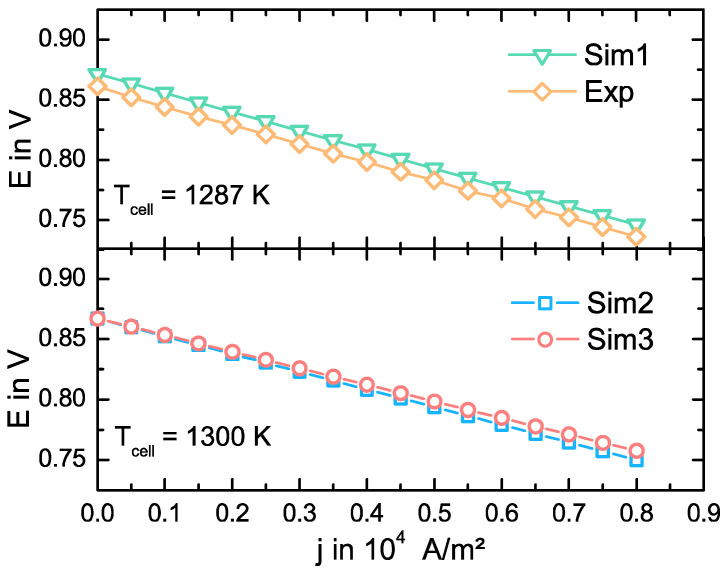
Experimental (Exp) and simulated voltage (E)-current(j)-characteristic (Sim1) at $T_{cell}=1287\;K$ (upper diagram), and simulated E-j-characteristics (Sim2 and Sim3) at $T_{cell}=1300\;K$ (lower diagram). We use the value $r^{e}\left(1287\;K\right)=0.0667\left(44\right)\;\Omega m$ in Sim1 and the value $r^{e}\left(1300\;K\right)=0.0622\left(40\right)\;\Omega m$ in Sim2. Both values are calculated from the experimental Arrhenius curve. In Sim3, we use the phenomenological coefficients for YSZ08 from Valadez Huerta et al. [40].

In Figure 4, we plot the measured (Exp) and the simulated E-j-characteristic (Sim1) for $T_{cell}=1287\;K$. We use in Sim1 the specific resistance $r^{e}\left(1287\;K\right)=0.0667\left(44\right)\;\Omega m$ calculated from the experimental Arrhenius curve and assume $L_{Oq}=L_{qO}=0$. The OCV value of the Sim1 curve deviates only 1.2% from the experimental value. It can be concluded from Equations (22) and (23) that gas leakages or uncertainties in the gas mixture concentration during the experiment may explain the deviation of the OCV to the theoretical value. Furthermore, losses due to a low electronic conductivity or a thermal gradient across the cell may also contribute to this deviation, since for $j=0\;A/m^{2}$ our model does not account such phenomena. We calculate the negative slope for each curve and assume it as the area specific resistance (ASR). The ASR is $1.5457\left(98\right)\times 10^{-5}\;{\Omega m}^{2}$ for the experimental curve and $1.5696\left(10\right)\times 10^{-5}\;{\Omega m}^{2}$ for the Sim1 curve. The relative deviation of the ASR between the experimental curve and Sim1 is only ~1.5%. Not only the losses due to the resistance in the electrolyte contribute to the ASR, but also the losses in the catalyst layer given by the reaction kinetics, as it can be taken from Equation (29). The low deviation means a successful implementation of Equations (26) and (27) using the experimental parameters for $\gamma^{i}$ and $E_{A,0}^{i}$, despite the high error bars in the activation energy $E_{A,j_{0}}^{a}$ and the pre-exponential factor $\gamma^{a}$. Furthermore, the relative deviation between the experimental voltage and the simulated voltage is only of ~1.3% for $j=8000\;A/m^{2}$. V. Eveloy et al. [54] reported model predictions within 3% to 6% of the cell voltage from different literature sources and, therefore, we can say that our simulation model effectively describes the E-j-characteristic for 1287 K.

To further validation, we calculate the E-j-characteristic using $r^{e}\left(1300\;K\right)=0.0622\left(40\right)\;\Omega m$ (Sim2) as well as the phenomenological coefficients for YSZ08 (Sim3) at 1300 K respectively and plot the results in Figure 4. From the discussion above, we can assume that the progression of the Sim2 curve may provide a satisfactory approach for the progression of the E-j-characteristic at 1300 K, because the simulation model, which is based on the same assumptions, effectively describes the E-j-progression for 1287 K. For that reason, we can use the Sim2 curve as a reference to evaluate the progression of the Sim3 curve. In this case and because of the same model framework, the OCV values are equal. The calculated ASR is $1.46524\left(80\right)\times 10^{-5}\;{\Omega m}^{2}$ for the Sim2 curve and $1.37117\left(80\right)\times 10^{-5}\;{\Omega m}^{2}$ for the Sim3 curve. The discrepancy between both ASRs of ~6.4% is mostly accounted by the relative deviation between the resistance calculated using $L_{OO}$ and the value from the experimental Arrhenius curve. Furthermore, the relative deviation of the voltage E between the Sim2 and the Sim3 curves is only ~1% for a current density of $8\times 10^{3}A/m^{2}$, meaning our model gives a satisfactory description of the cell up to this current density.

### 3.3. Simulation Results

**Figure 5:**
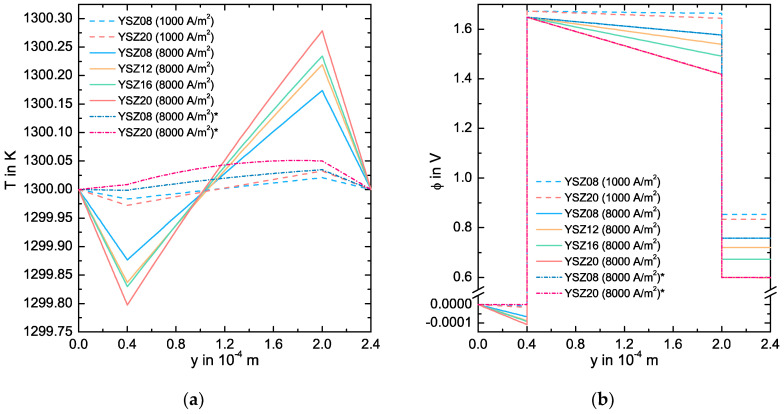
(**a**) Simulated temperature and (**b**) electric potential profile across the SOFC at 1300 K for various YSZ-electrolytes and the current densities $j=1000\;A/m^{2}$ and $j=8000\;A/m^{2}$. For the simulated curves marked with *, we neglect all Peltier type coupled mechanisms.

In Figure 5a, the simulated temperature profile across the SOFC at 1300 K is depicted for various YSZ-electrolytes and $j=1000\;A/m^{2}$ and $8000\;A/m^{2}$. The temperature gradient is negative in the GDLs and positive in the electrolyte. In all cases, the progression of the temperature can be seen as linear. A higher current density and a higher electrolyte Y_2_O_3_ concentration result in higher absolute values for the temperature gradient in all of the bulk phases. Furthermore, a minimum at the anode and a maximum at the cathode can be observed. To explain these effects, we plot in Figure 5a the results of a simulation for YSZ08 and YSZ20 at $j=8000\;A/m^{2}$, where we *neglect* all Peltier (coupling) mechanisms. Here, all absolute values of the temperature gradients are lower than for the original conditions. In the YSZ20 electrolyte case, the one in the anode is positive and the one in the electrolyte does not progress linearly, experiencing a maximum at $y=1.8\times 10^{-4}\;m$. From a comparison with the previous results, the high absolute values for the temperature gradients may result from a Seebeck mechanism. While $J_{O^{-2}}^{e}$, $L_{Oq}$ and $z^{*}$ are negative in Equation (41), $L_{OO}$ is positive. A high potential loss in the electrolyte results in a high negative value of the potential gradient and the negative Ohmic term decreases. To counter this and to assure a constant anion flux, the temperature gradient has to have a high positive value. This may explain not only the pronounced temperature progression, but also its dependency on the current density, since a higher anion flux gives a higher counter effect. A calculation without these coupled mechanisms results in a significantly different temperature progression.

In Figure 5b, we plot the profile of the electric potential across the cell. In all bulk phases, the potential decreases. The progression also shows two jumps: a positive at the anode CL and a negative at the cathode CL. These jumps result from the equilibrium potentials $\Delta \phi_{0}^{a}=-1.68\;V$ and $\Delta \phi_{0}^{c}=-0.81\;V$, and from the activation losses in each electrode. We calculate the over-potentials $\eta^{a}=3.4\cdot 10^{-3}\;V$ and $\eta^{c}=-9.3\times 10^{-4}\;V$ for $1000\;A/m^{2}$, and $\eta^{a}=0.027\;V$ and $\eta^{c}=-7.3\times 10^{-3}\;V$ for $8000\;A/m^{2}$. As expected, $\eta^{a}$ is positive, $\eta^{c}$ is negative and their magnitude does not depend on the electrolyte type. The potential losses in the bulk phases increase for a higher current density and for a higher Y_2_O_3_ concentration in the electrolyte. Since the potential losses in the anode GDL and in the cathode GDL are too small, the progression of the curve is mostly defined by the losses in the electrolyte. To give a more detailed discussion, Figure 5b also includes results for simulations neglecting all Peltier mechanisms. The progression of the potential in the electrolyte is not substantially different from the original results and the losses are mainly Ohmic. However, the potential losses in the GDLs are mainly given by the temperature gradient rather than by the electric resistance. To justify this statement, we can argue analogous to the discussion of the pronounced temperature gradient by using Equation (12). We can calculate the effective specific resistance $r^{e}\left(1300\;K\right)\approx 0.05621\;\Omega m$ using the potential gradient across the electrolyte and the current density. This value deviates only ~0.2% from the one calculated with the conductivity $L_{OO}$. Therefore, the potential losses due to the coupled effects do not significantly affect the progression of the E-j charactersitic and the direct comparison between $L_{OO}$ and $r^{e}$ in Section 3.1 is appropriate.

**Figure 6:**
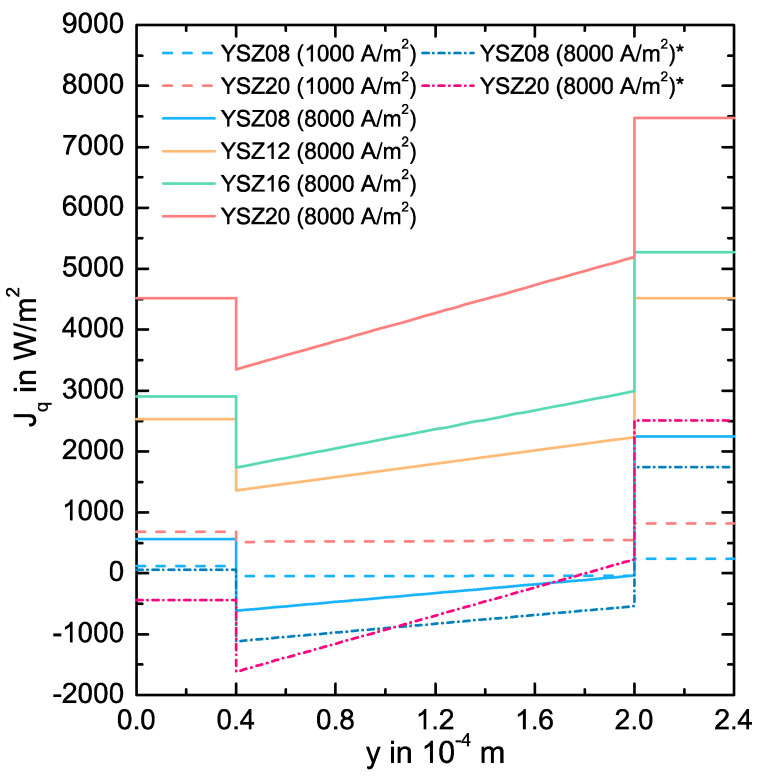
Simulated heat flux $J_{q}\left(y\right)$ across the SOFC at 1300 K for various YSZ-electrolytes and the current densities $j=1000\;A/m^{2}$ and $j=8000\;A/m^{2}$. For the simulated curves marked with *, we neglect all Peltier type coupled mechanisms.

We plot in Figure 6 the heat flux $J_{q}$ across the cell. We only calculate the net heat flux in the y-direction. It increases in the electrolyte with a slope $dJ_{q}^{e}/dy$, which becomes higher for higher current densities and higher potential losses due to energy conservation (see Equation (43)). Furthermore, it remains almost constant at the GDLs. The heat flux shows a negative jump at the anode CL and a positive jump at the cathode CL, meaning the anode half reaction is endothermic and the cathode half reaction is exothermic. This is in agreement with the study of K. Fischer and J. R. Seume [55]. Both jumps increase for a higher current density due to the higher activation losses, yet they remain almost the same for the different electrolytes. Independent of the current density, the heat flows across the anode GDL and across the cathode GDL in y-direction for YSZ08, but across the electrolyte opposite to this direction. As expected from classical behavior, the heat flows from a higher to a lower temperature. However, the heat is transported from a low to a high temperature in electrolytes with a higher Y_2_O_3_ concentration, contradicting Fourier conduction behavior. In Figure 6, the heat flux resulting from a simulation without any coupled effects is given for the cases of an YSZ08 and an YSZ20 electrolyte at $8000\;A/m^{2}$. Due to energy conservation, the progression of these curves is almost equal to the progression of the original curves. However, the amount and sign of the heat flux is different. If the coupled mechanisms are considered, the heat transported is higher in all layers. On one hand, the higher heat flux at the GDLs results from the higher temperature gradient, since the Peltier-effect may reduce it (see Equation (11)). On the other hand, the higher heat flux in the electrolyte results from the potential gradient, since the positive temperature gradient may diminish it (see Equation (40)). This effect is most pronounced for a higher Y_2_O_3_ concentration in the electrolyte and a higher current density due to the higher potential losses. As it can be seen from the YSZ20 case, if the coupled mechanisms are neglected, not only the amount of heat transported may be underestimated, but also the direction of the heat flux. This may lead to the wrong decisions regarding the material design or heating/cooling strategies of SOFCs, such as which gas inlet flow may be used for cooling the cell to minimize thermal stress.

**Figure 7:**
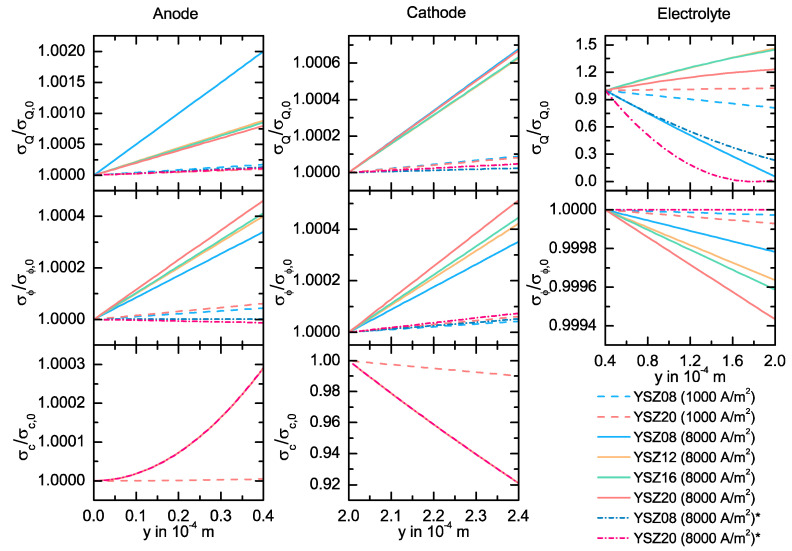
Simulated local entropy production rate contributions ${\dot{\sigma }}_{Q}$ (heat flux contribution), ${\dot{\sigma }}_{\phi }$ (potential contribution) and ${\dot{\sigma }}_{c}$ (diffusion contribution) in the anode GDL, in the cathode GDL and in the electrolyte. We normalized the values with ${\dot{\sigma }}_{i,0}={\dot{\sigma }}_{i}\left(y=0\right)$ for the anode, ${\dot{\sigma }}_{i,0}={\dot{\sigma }}_{i}\left(y=2\times 10^{-4}\;m\right)$ for the cathode and (c) ${\dot{\sigma }}_{i,0}={\dot{\sigma }}_{i}\left(y=0.4\times 10^{-4}m\right)$ for the electrolyte (see Table 3). The curves are given for a SOFC at 1300 K, for various YSZ-electrolytes and two current densities, $j=1000\;A/m^{2}$ and $j=8000\;A/m^{2}$. For the simulated curves marked with *, we neglect all Peltier type coupled mechanisms.

In Figure 7, the heat contribution $\dot{\sigma_{Q}}$, the potential contribution ${\dot{\sigma }}_{\phi }$ and the diffusion contribution ${\dot{\sigma }}_{c}$ to the local entropy production rate $\dot{\sigma }$ are depicted for each layer for different electrolyte compositions at $1000\;A/m^{2}$ and $8000\;A/m^{2}$. $\dot{\sigma_{Q}}$ is the term given by the temperature gradient (see Equations (20) and (42)), ${\dot{\sigma }}_{\phi }$ the term given by the potential gradient (see Equations (20) and (42)), and ${\dot{\sigma }}_{c}$ the concentration dependent term (see Equations (20)–(22)). We also plot two simulation results neglecting all Peltier mechanisms. We normalize all terms with the value ${\dot{\sigma }}_{i,0}$ at the beginning of each layer, because the magnitude of the values is of a different order for each case (see Table 3) and, thus, a direct comparison is difficult.

The absolute values for ${\dot{\sigma }}_{Q,0}$ in all layers become higher for increasing current density and increasing Y_2_O_3_ content. Due to the Peltier effect, most of the values are negative across the electrolyte. These negative contributions are smaller than the potential contribution and a positive local entropy production rate is always ensured. The heat flux contributions are only positive for YSZ08 and, clearly, for the cases assuming no Peltier effect. For these latter cases, the absolute values for ${\dot{\sigma }}_{Q,0}$ are smaller than for the original cases. The values for ${\dot{\sigma }}_{Q,0}$ are proportional to the temperature gradient (see Equations (20) and (42)) and, thus, all these dependencies can be explained following the above discussion. The absolute values for ${\dot{\sigma }}_{Q,0}$ are higher across the GDLs than across the electrolyte in each case, resembling the results for the heat flux in Figure 6. Looking at Figure 7, the value of the heat flux contribution ${\dot{\sigma }}_{Q}$ in the GDLs increases slightly in the y-direction. If we assume a constant temperature gradient, the temperature and the heat flux progression may define the progression of ${\dot{\sigma }}_{Q}$. Here, the dependency on the Y_2_O_3_ concentration is not well defined and, thus, we cannot find a direct correlation to the curves for the temperature or the heat flux. Therefore, the resulting behavior may be given from a combination of both effects. This may also explain the low changes in the progression of the curves for lower current densities and for the cases neglecting all coupled mechanisms. However, these changes are smaller than 0.2% of the initial value. As already discussed, the negative potential gradient in the electrolyte and a high current density result into an increasing heat flux due to energy conservation. Together with the increasing temperature appearing in the denominator of the term ${\dot{\sigma }}_{Q}$ in Equation (43), the negative progression of $J_{q}^{e}$ results in a decreasing progression of ${\dot{\sigma }}_{Q}$ for YSZ08. This trend changes for higher Y_2_O_3_ concentrations because the heat flux becomes increasing positive due to the Peltier effect. This behavior is observed for both current densities studied here. For an YSZ08 electrolyte, the value can decrease by 30% across the electrolyte and, for higher compositions, the value can increase by 150% depending on the current density. Additionally, the progression for ${\dot{\sigma }}_{Q}$ is not well predicted if the coupled effects are neglected, resulting in a strong decaying tendency independent of the electrolyte.

The values ${\dot{\sigma }}_{\phi ,0}$ increase for higher current density and higher Y_2_O_3_ composition in all bulk phases, but its dependency on the electrolyte composition is not given in the GDLs if the Peltier effect is neglected. The dependency on the current density and on the Y_2_O_3_ composition is given by Equation (20), where ${\dot{\sigma }}_{\phi ,0}$ is negatively proportional to the product between j and the potential gradient divided by the temperature. The low values for ${\dot{\sigma }}_{\phi ,0}$ in the GDLs for the cases assuming no Peltier effect result from the low potential gradients. As expected from the similar progression of the potential with and without any Peltier mechanisms, all ${\dot{\sigma }}_{\phi ,0}$ values in the electrolyte are similar for both cases. Furthermore, this contribution is the highest across the cell. For all curves in Figure 7, the slightly increasing or decreasing progression of ${\dot{\sigma }}_{\phi }$ is given by the temperature. This statement is justified, since the dependency on the spatial dimension, on the current density, on the Y_2_O_3_ composition and on whether the Peltier mechanisms are taken into account, is in agreement with the results depicted in Figure 5.

As expected from Equations (21) and (22), the values of ${\dot{\sigma }}_{c,0}$ depend only on the current density and not on the YSZ composition of the electrolyte. Since the temperature and potential differences at the GDLs are very low, the diffusion process provides the major contribution to the local entropy production rate. This contribution is lower at the anode GDL than at the cathode GDL. Here, the losses due to the oxygen diffusion are higher than the losses due to the diffusion of hydrogen and water at the anode GDL. The non-linearity of the increasing progression of ${\dot{\sigma }}_{c}$ at the anode can be accounted for by the superposition of a decreasing hydrogen concentration and an increasing water concentration across the GDL. This effect is not present at the cathode GDL, where the increasing oxygen concentration in the y-direction results in a decreasing progression of ${\dot{\sigma }}_{C}$.

The jump in the entropy production rate per area at the anode CL is $2.6\times 10^{-3}\;W/m^{2}K$ for $1000\;A/m^{2}$ and $0.17\;W/m^{2}K$ for $8000\;A/m^{2}$, and at the cathode CL $7.1\times 10^{-4\;}W/m^{2}K$ for $1000\;A/m^{2}$ and $0.045\;W/m^{2}K$ for $8000\;A/m^{2}$. These jumps do not depend substantially on the Y_2_O_3_ concentration of the electrolyte. To make a comparison between the entropy production at the CLs and at the bulk phases, non-specific values are needed. If we consider an YSZ12 cell with a 4 cm × 4 cm active area working at $8000\;A/m^{2}$ and use the values given in Table 3 as an estimate for the entropy production rate contributions, it results in an entropy production rate of $4.60\times 10^{-5}\;W/K$ in the GDLs, of $1.08\times 10^{-3}\;W/K$ in the electrolyte, and of $3.44\times 10^{-4}\;W/K$ at the CLs. This means that 3% of the losses would originate in the GDLs, 73% in the electrolyte, and 24% at the CLs. These values are expected for an ESC like the one studied here.

## 4. Summary and Conclusions

The main goal of this study is the 1D-calculation of the local entropy production rate across an electrolyte supported planar SOFC at 1300 K and atmospheric pressure. We describe all bulk phases using non-equilibrium thermodynamics. While we use empirical coefficients from the literature to describe the GDLs, the phenomenological coefficients from our previous MD study [40] are used to characterize the cell with various electrolytes of different Y_2_O_3_ content. We describe the CLs using the Butler–Volmer ansatz. We conduct EIS measurements in a YSZ08 SOFC to derive the parameters to describe the exchange current densities $j_{0}^{i,0}$. Moreover, we measure the E-j-characteristic and use this data to validate our model. Here, we show that the Butler–Volmer kinetics is well parametrized and implemented. Furthermore, the relative deviation of ~6.4% between the ASR of both curves is mainly due to the deviation of the electrolyte resistance calculated with $L_{OO}$ to the experimental value of ~10%. For a current density of $8000\;A/m^{2}$ the relative deviation between both E-j-characteristics is only ~1%, so that our approach effectively describes the SOFC. The temperature gradient across each layer is higher for a higher Y_2_O_3_ electrolyte concentration and a higher current density. This effect results from the coupled mechanisms and, thus, if the Peltier effect is neglected, the temperature profile across the cell is not properly described. From the potential profile, we conclude that the highest contribution to the electric potential losses are in the electrolyte. These losses are higher for a higher Y_2_O_3_ concentration and no substantial differences arise, if the simulation does not consider any coupled effects. Using the entropy balance for the calculation of the heat flux at the CLs, it results in an endothermic half reaction at the anode and an exothermic half reaction at the cathode. Moreover, the description of the transport mechanisms across a SOFC using NET cannot be avoided, since the heat flux in the electrolyte is mainly given by the Peltier effect. If this effect is neglected, the heat flux will not be properly described. Finally, we calculate the heat flux contribution, the potential contribution, and the diffusion contribution to the local entropy production rate. The entropy production rate across the electrolyte is mostly given by the ionic conduction, since the Peltier heat transfer from lower to higher temperature would reduce the entropy production. While the temperature and the heat flux define the progression of ${\dot{\sigma }}_{Q}$ across the electrolyte, the potential contribution profile is mainly given by the temperature. Despite the appreciable increasing or decreasing tendency of the contributions to the entropy production rate, a substantial change along the y-direction can be observed only for the heat flux contribution in the electrolyte. If we neglect the Peltier effect, the values for the heat flux and potential contribution are underestimated in the GDLs and a different profile of the heat flux contribution to the local entropy production rate is predicted across the electrolyte. Finally, the entropy production rate of a cell is the highest in the electrolyte (73%) followed by the contribution in both GDLs with (24%).

## Figures and Tables

**Table 1:** Parameters used in the simulation for the GDLs and the CLs. Values taken from Hajimolana et al. [25], Kjelstrup and Bedeaux [20], and Costamagna et al. [24].

Parameter	Anode GDL	Anode CL	Cathode CL	Cathode GDL	Reference
$\Delta y_{i}$ in $10^{-6}\;m$	40	-	-	40	-
$r_{0}^{i}$ in $10^{-6}\;\Omega m/K$	1/95	-	-	1/42	[25]
$E_{A,r}^{i}/R$	1150	-	-	1200	[25]
$\lambda^{i}$ in W/mK	2	-	-	2	[20]
$\pi^{i}/T_{cell}$ in J/(CK)	$-5.4\times 10^{-4}$	-	-	$-6.2\times 10^{-4}$	[20]
$D_{H_{2}}=D_{H_{2}O}$ in $m^{2}/s$	$2.1\times 10^{-5}$		-	-	[24]
$D_{O_{2}}$ in $m^{2}/s$	-	-	$5.4\times 10^{-6}$		[24]
$\alpha^{i}$	-	0.5	0.3	-	[24]

**Table 2:** Parameters used in the phenomenological equations to describe the electrolyte. The values are taken from the MD study of Valadez Huerta et al. [40].

YSZ	$L_{OO}$ $\;\mathrm{in}\;10^{-7}mol^{2}K{\left(\mathrm{Jms}\right)}^{-1}$	$L_{qq}$ $\;\mathrm{in}\;10^{6}\;W/m$	$RL_{oq}$ in W/m
YSZ08	6.22(79)	6.21(25)	−0.81(69)
YSZ12	4.10(57)	5.37(27)	−0.77(75)
YSZ16	2.85(36)	4.85(34)	−0.55(56)
YSZ20	1.95(55)	4.54(29)	−0.48(36)

**Table 3:** Values of the entropy production rate used for the normalization in Figure 7. For the values marked with *, we neglect all Peltier type coupled mechanisms.

YSZ|j	Anode GDL ${\dot{\sigma }}_{i}\left(y=0\;m\right)$ $\;W/\left(m^{2}K\right)$ i=Q|ϕ|C	Electrolyte ${\dot{\sigma }}_{i}\left(y=0.4\times 10^{-4}m\right)$ $W/\left(m^{2}K\right)$ i=Q|ϕ	Cathode GDL ${\dot{\sigma }}_{i}\left(y=2\times 10^{-4}m\right)$ $W/\left(m^{2}K\right)$ i=Q|ϕ|C
YSZ08|$1000\;A/m^{2}$	0.03|0.17|4.60	0.007|43.24	0.074|0.25|5.37
YSZ20|$1000\;A/m^{2}$	0.28|0.29|4.60	−0.12|138.6	0.40|0.39|5.37
YSZ08|$8000\;A/m^{2}$	1.02|10.26|294.20	0.70|2767	5.77|16.58|369.73
YSZ12|$8000\;A/m^{2}$	6.11|13.54|294.20	−2.04|4204	14.65|20.91|369.73
YSZ16|$8000\;A/m^{2}$	7.33|14.16|294.20	−2.82|6033	18.27|22.35|369.73
YSZ20|$8000\;A/m^{2}$	13.55|16.84|294.20	−6.64|8871	30.77|26.55|369.73
YSZ08*|$8000\;A/m^{2}$	0.001|0.001|294.20	0.20|2760	0.90|0.003|369.73
YSZ20*|$8000\;A/m^{2}$	0.06|0.001|294.20	0.57|8846	1.86|0.003|369.73
